# Development of an oncological-multidimensional prognostic index (Onco-MPI) for mortality prediction in older cancer patients

**DOI:** 10.1007/s00432-015-2088-x

**Published:** 2016-01-12

**Authors:** Antonella Brunello, Andrea Fontana, Valeria Zafferri, Francesco Panza, Pasquale Fiduccia, Umberto Basso, Massimiliano Copetti, Sara Lonardi, Anna Roma, Cristina Falci, Silvio Monfardini, Alberto Cella, Alberto Pilotto, Vittorina Zagonel

**Affiliations:** 1 Department of Clinical and Experimental Oncology, Medical Oncology 1, Istituto Oncologico Veneto IOV – IRCCS, Padua, Italy; 2 IRCCS Casa Sollievo della Sofferenza, San Giovanni Rotondo, Foggia, Italy; 3 Neurodegenerative Disease Unit, Department of Basic Medicine, Neuroscience, and Sense Organs, University of Bari Aldo Moro, Bari, Italy; 4 Medical Oncology 2, Istituto Oncologico Veneto IOV- IRCCS, Padua, Italy; 5 IRCCS Fondazione Don Gnocchi, Milan, Italy; 6 Department of OrthoGeriatrics, Rehabilitation and Stabilization, Frailty Area, NR-HS Galliera Hospital, Genoa, Italy; 7 Geriatrics Unit, Azienda ULSS 16, S Antonio Hospital, Padua, Italy

**Keywords:** Cancer, Elderly, Prognosis, Mortality, Multidimensional prognostic index (MPI), Comprehensive geriatric assessment (CGA)

## Abstract

**Purpose:**

A multidimensional prognostic index (MPI) based on a comprehensive geriatric assessment (CGA) has been developed and validated in independent cohorts of older patients demonstrating good accuracy in predicting one-year mortality. The aim of this study was to develop a cancer-specific modified MPI (Onco-MPI) for mortality prediction in older cancer patients.

**Methods:**

We enrolled 658 new cancer subjects ≥70 years (mean age 77.1 years, 433 females, 65.8 %) attending oncological outpatient services from September 2004 to June 2011. The Onco-MPI was calculated according to a validated algorithm as a weighted linear combination of the following CGA domains: age, sex, basal and instrumental activities of daily living, Eastern Cooperative Oncology Group performance status, mini-mental state examination, body mass index, Cumulative Illness Rating Scale, number of drugs and the presence of caregiver. Cancer sites (breast 46.5 %, colorectal 21.3 %, lung 6.4 %, prostate 5.5 %, urinary tract 5.0 %, other 15.3 %) and cancer stages (I 37 %, II 22 %, III 19 %, IV 22 %) were also included in the model. All-cause mortality was recorded. Three grades of severity of the Onco-MPI score (low risk: 0.0–0.46, medium risk: 0.47–0.63, high risk: 0.64–1.0) were calculated using RECPAM method. Discriminatory power and calibration were assessed by estimating survival C-indices, along with 95 % confidence interval (CI) and the survival-based Hosmer–Lemeshow (HL) measures.

**Results:**

One-year mortality incidence rate was 17.4 %. A significant difference in mortality rates was observed in Onco-MPI low risk compared to medium- and high-risk patients (2.1 vs. 17.7 vs. 80.8 %, *p* < 0.0001). The discriminatory power of one-year mortality prediction of the Onco-MPI was very good (survival C-index 0.87, 95 % CI 0.84–0.90) with an excellent calibration (HL *p* value 0.854).

**Conclusion:**

Onco-MPI appears to be a highly accurate and well-calibrated predictive tool for one-year mortality in older cancer patients that can be useful for clinical decision making in this age group.

## Introduction

In both Europe and in the USA, the majority of persons who receive a cancer diagnosis are aged 65 years or older (Siegel et al. 2012), and the number of older patients with cancer is expected to rise substantially in the next decades (Smith et al. 2009). One of the priorities for both clinicians and researchers is the assessment, treatment planning and evaluation of outcomes of these subjects. However, ongoing enrollment biases with underrepresentation of older individuals in clinical trials of cancer (Talarico et al. 2004; Scher and Hurria 2012) greatly limit that evidence-based clinical decisions be taken in such a population. Particularly, determining life expectancy related to functional status and comorbidity seems to be of utmost importance in that it could help in moving beyond arbitrary age-based cutoffs when making decisions of treating older patients with chemotherapy both in the adjuvant setting and for advanced disease (Gill 2012). Several studies demonstrated that in older subjects, the final prognosis is linked to multimorbidity and multidimensional impairment, i.e., an impairment in the functional, cognitive, nutritional and psychosocial domains (Yourman et al. 2012), that are appropriately explored at their best by using a comprehensive geriatric assessment (CGA) also in older patients with cancer (Caillet et al. 2014). Indeed, while recent guidelines recommend life expectancy inclusion in clinical decision-making paths in older age (Pilotto et al. 2015), at present, no validated CGA-based prognostic instruments are available to guide treatment plan in older cancer patients (Baijal and Periyakoil 2014).

Recently, a multidimensional prognostic index (MPI) has been developed and validated on the basis of a standardized CGA (Pilotto et al. 2008), which take into account eight domains related to functional and cognitive status, nutrition, comorbidities, pressure sore risk, number of medications and social status. The MPI has been shown to be an accurate predictor for short- and long-term mortality in patients hospitalized for acute or reactivation of chronic diseases such as community-acquired pneumonia, dementia, congestive heart failure, chronic kidney disease, and other most common disorders leading to death in the elderly (Pilotto et al. 2012a, b, c) as well as in hospitalized patients with cancer (Giantin et al. 2013). The aim of this study was to develop a cancer-specific MPI (Onco-MPI) applicable in the outpatient setting to predict mortality in older patients with different types of cancer, so recognizing heterogeneity in this age group and leading to individualized approaches toward cancer treatment.

## Methods

### Study population

Patients aged ≥70 referred to the Geriatric Oncology Program of the Istituto Oncologico Veneto (IOV) in Padova, Italy, from September 2004 to June 2011 were included. Patients needed to have a confirmed diagnosis of cancer and a complete CGA recorded in the clinical chart to be included. For all patients, the following variables were collected: age, gender, Eastern Cooperative Oncology Group (ECOG) performance status (Oken et al. 1982), associated diseases and their severity graded according to Cumulative Illness Rating Scale (CIRS) (Linn et al. 1968), present medications, the presence of pain, body mass index (BMI), site and stage of primary cancer, cancer treatment planned and/or received, living status/the presence of caregiver, basal and instrumental activities of daily living (ADL, IADL) (Katz et al. 1970; Lawton and Brody. 1969), mini-mental state examination (MMSE) (Folstein et al. 1975) and the 15-item Geriatric Depression Scale (GDS) (Satin et al. 2009).

All patients were followed up with clinical visits every 3–6 months with a median follow-up time of 2.5 years and a range of 0.0–8.2 years. For patients who died during the follow-up, the date of death was obtained from clinical charts when available, and it was collected either from death certificates or by contacting demographic offices when not available. For subjects who did not experience the end point, survival time was censored at the time of the last available follow-up visit.

### The oncological-multidimensional prognostic index (Onco-MPI)

To build the Onco-MPI, a weighted sum of the following domains was computed (raw formula): age, sex, ADL, IADL, ECOG performance status, MMSE, BMI, CIRS, number of drugs, the presence of caregiver, cancer sites and cancer stages. Weights were estimated from a multivariable Cox proportional hazard model, within 1 year of follow-up. Each weighted sum was then normalized into a range that varies from 0 (lowest risk) to 1 (highest risk), subtracting the observed raw minimum value (i.e., −2.371) and then dividing such difference by the observed range (minimum to maximum span, i.e., 8.034). Three grades of Onco-MPI severity were estimated using RECursive Partition and AMalgamation (RECPAM) algorithm. At each partitioning step, the method chooses the best binary split (cutoff) to maximize the difference in the outcome of interest. Discriminatory power was assessed by estimating survival C-indices, along with 95 % confidence interval (CI) (Pencina and D’Agostino 2004), and the survival-based Hosmer–Lemeshow (HL) measure of calibration (D’Agostino and Nam 2004) was also assessed.

### Statistical analysis

Patients’ baseline characteristics were reported as mean ± standard deviation (SD) or frequencies and percentage for continuous and categorical variables, respectively. Medians and ranges were reported for continuous variables. The overall survival was defined as the time between date of first visit and death. Mortality incidence rates were reported as the observed number of events for 100 person-years and were compared using a Poisson model. Time-to-death analyses were performed using univariate and multivariate Cox regression models, within 1 year of follow-up, and results were expressed as hazard ratios (HRs) and 95 % CI. Survival curves were reported according to the Kaplan–Meier method.

All statistical analyses were performed using SAS version 9.3 (SAS Institute, Cary, NC).

## Results

Of all older patients ≥70 years referred for evaluation as new patients to the Geriatric Oncology Program from September 2004 to June 2011, full CGA data were available for 658 patients. Baseline patients’ characteristics are shown in Table 1.

**Table 1:** Baseline patients’ characteristics

Variable	Category	
No of patients		658
Age (years)	Mean ± SD	77.16 ± 5.11
	Median (min–max)	77.00 (70.00–96.00)
Sex (*n*, %)	Females	433 (65.81 %)
	Males	225 (34.19 %)
BMI (kg/m^2^)	Mean ± SD	25.49 ± 4.21
	Median (min–max)	25.22 (15.50–47.56)
ADL	Mean ± SD	5.71 ± 0.85
	Median (min–max)	6.00 (0.00–6.00)
IADL	Mean ± SD	6.83 ± 1.80
	Median (min–max)	8.00 (0.00–8.00)
The presence of comorbidity (*n*, %)	No	495 (75.23 %)
	Yes	163 (24.77 %)
Comorbidity index CIRS	Mean ± SD	1.71 ± 1.29
	Median (min–max)	2.00 (0.00–7.00)
No of total comorbidities CIRS	Mean ± SD	2.72 ± 1.60
	Median (min–max)	3.00 (0.00–9.00)
No of severe comorbidities CIRS	Mean ± SD	0.31 ± 0.60
	Median (min–max)	0.00 (0.00–4.00)
MMSE (*n*, %)	<24	119 (18.09 %)
	≥24	539 (81.91 %)
The presence of psychiatric disease (*n*, %)	No	606 (92.10 %)
	Yes	52 (7.90 %)
Cancer stage (*n*, %)	I	246 (36.39 %)
	II	144 (21.88 %)
	III	121 (18.39 %)
	IV	147 (22.34 %)
Cancer treatment (*n*, %)	No	160 (24.32 %)
	Yes	498 (75.68 %)
No of drugs	Mean ± SD	3.32 ± 2.43
	Median (min–max)	3.00 (0.00–13.00)
ECOG performance status	Mean ± SD	0.52 ± 0.72
	Median (min–max)	0.00 (0.00–4.00)
Caregiver (*n*, %)	No	193 (29.33 %)
	Yes	465 (70.67 %)
15-item Geriatric Depression Scale (*n*, %)	≤ 5	497 (75.53 %)
	> 5	161 (24.47 %)
Geriatric syndromes (*n*, %)	No	609 (92.55 %)
	Yes	49 (7.45 %)
Tumor site (*n*, %)	Breast	306 (46.50 %)
	Colorectal	140 (21.28 %)
	Lung	42 (6.38 %)
	Prostate	36 (5.47 %)
	Other genitourinary	33 (5.02 %)
	Other	101 (15.35 %)

*BMI* body mass index, *ADL* activities of daily living, *IADL* instrumental activities of daily living, *CIRS* Cumulative Illness Rating Scale, *MMSE* mini-mental state examination, *ECOG* Eastern Cooperative Oncology Group

After a median follow-up time of 2.5 years (range 0.0–8.2), 105 patients died, with an overall one-year mortality incidence rate of 17.4 %. Univariate Cox regressions analysis showed that 11 domains of the CGA were significantly associated with mortality (Table 2).

**Table 2:** Results from univariable Cox regressions for mortality risk prediction, within 1 year of follow-up in older cancer patients

Variable	Category	HR (95 % CI)	*p* value
Age	Cont. Var.	1.076 (1.039–1.114)	<0.001
Sex	Male versus female	2.084 (1.422–3.056)	<0.001
BMI	Cont. Var.	0.870 (0.824–0.918)	<0.001
ADL	Cont. Var.	0.667 (0.582–0.764)	<0.001
IADL	Cont. Var.	0.765 (0.709–0.825)	<0.001
The presence of comorbidity	Yes versus no	1.420 (0.941–2.145)	0.095
Comorbidity Index CIRS	Cont. Var.	1.011 (0.873–1.173)	0.880
No of total comorbidites CIRS	Cont. Var.	1.036 (0.921–1.164)	0.557
No of severe comorbidites CIRS	Cont. Var.	1.288 (0.985–1.685)	0.065
MMSE	<24 versus ≥24	1.913 (1.253–2.922)	0.003
Psychiatric diseases	Yes versus no	0.809 (0.376–1.742)	0.589
Cancer stage	IV versus I	17.089 (8.189–35.659)	<0.001
	III versus I	4.555 (1.966–10.555)	<0.001
	II versus I	3.614 (1.546–8.443)	0.003
Cancer treatment	Yes versus no	0.726 (0.478–1.105)	0.135
No of drugs	Cont. Var.	1.036 (0.960–1.117)	0.363
Performance status (continuous)	Cont. Var.	2.133 (1.766–2.576)	<0.001
ECOG performance status (categorical)	≥3 versus 0	6.184 (2.163–17.679)	<0.001
	2 versus 0	7.418 (4.223–13.031)	<0.001
	1 versus 0	4.232 (2.658–6.738)	<0.001
Caregiver	Yes versus no	1.603 (1.002–2.565)	0.049
15-item Geriatric Depression Scale	>5 versus ≤5	1.402 (0.926–2.125)	0.111
Syndromes	Yes versus no	1.329 (0.693–2.550)	0.392
Tumor site	Breast versus other	0.078 (0.038–0.157)	<0.001
	Colorectal versus other	0.401 (0.234–0.685)	<0.001
	Lung versus other	1.429 (0.815–2.506)	0.213
	Prostate versus other	0.335 (0.131–0.856)	0.022
	Other genitourinary versus other	1.372 (0.747–2.520)	0.308

*BMI* body mass index, *ADL* activities of daily living, *IADL* instrumental activities of daily living, *CIRS* Cumulative Illness Rating Scale, *MMSE* mini-mental state examination, *ECOG* Eastern Cooperative Oncology Group, *Cont. Var.* continuous variable

In detail, one-year mortality risk was associated with increasing age, male sex, lower MMSE, impaired ADL, impaired IADL, number of severe comorbidities according to CIRS, poor ECOG performance status, the presence of caregiver and late-stage cancer, whereas having a breast cancer diagnosis (vs. all other cancers) and higher BMI predicted lower mortality.

As shown in Table 3, the weights used to build the Onco-MPI were the regression coefficients (logarithm of the HR) estimated from a multivariate Cox model (Table 4). After the normalization procedure, our score ranged from 0 (low risk) to 1 (high risk).

**Table 3:**
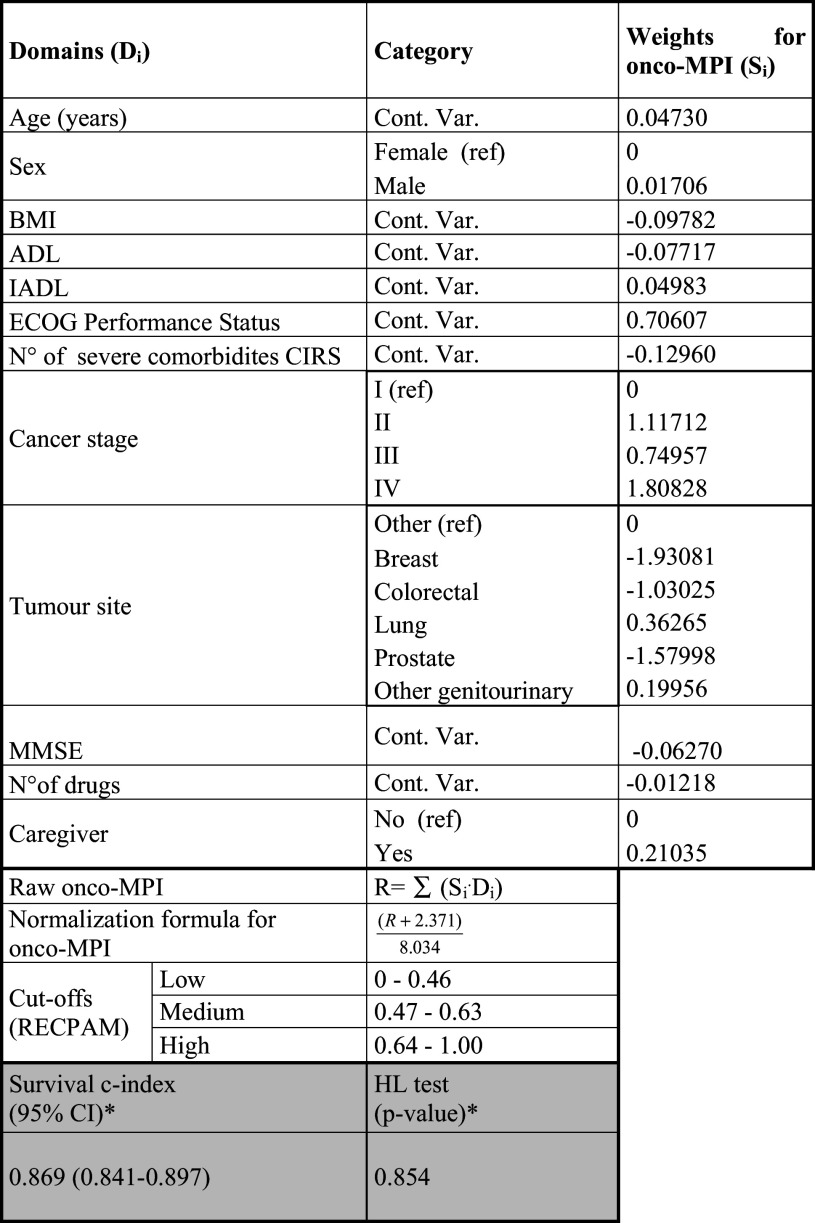
Estimated domains weights used to compute the onco-multidimensional prognostic index (MPI), for mortality risk prediction within 1 year of follow-up

Defined using: age, sex, *BMI* body mass index, *ADL* activities of daily living, *IADL* instrumental activities of daily living, *ECOG* Eastern Cooperative Oncology Group performance status, *CIRS* Cumulative Illness Rating Scale, *MMSE* mini-mental state examination, number of drugs, the presence of a caregiver, cancer stage and tumor size

*Cont. Var.* continuous variable

* Survival C-index, along with 95 % confidence intervals (CI), and *p* value from Hosmer–Lemeshow (HL) goodness-of-fit test for calibration of the Onco-MPI score within 1 year of follow-up

**Table 4:** Results from multivariable Cox regressions for mortality risk prediction, within 1 year of follow-up in older cancer patients

Variable	Category	HR (95 % CI)	*p* value
Age	Cont. Var.	1.040 (0.996–1.086)	0.075
Sex	Male versus female	1.018 (0.647–1.600)	0.939
BMI	Cont. Var.	0.912 (0.859–0.968)	0.002
ADL	Cont. Var.	0.920 (0.751–1.128)	0.423
IADL	Cont. Var.	1.008 (0.870–1.167)	0.92
Comorbidity index CIRS	Cont. Var.	0.914 (0.749–1.114)	0.372
MMSE	<24 versus ≥24	0.971 (0.595–1.584)	0.906
Psychiatric diseases	Yes versus no	0.498 (0.198–1.251)	0.138
Cancer stage	IV versus I	6.689 (2.950–15.166)	<0.001
	III versus I	2.129 (0.854–5.306)	0.105
	II versus I	3.335 (1.389–8.009)	0.007
Cancer treatment	Yes versus no	0.984 (0.618–1.567)	0.945
No of drugs	Cont. Var.	1.023 (0.923–1.133)	0.668
ECOG performance status	≥3 versus 0	7.747 (1.744–34.411)	0.007
	2 versus 0	3.827 (1.777–8.244)	<0.001
	1 versus 0	3.122 (1.877–5.191)	<0.001
Caregiver	Yes versus no	1.193 (0.708–2.010)	0.508
15-item Geriatric Depression Scale	>5 versus ≤5	0.947 (0.574–1.564)	0.833
Syndromes	Yes versus no	1.050 (0.494–2.234)	0.898
Tumor site	Breast versus other	0.164 (0.068–0.396)	<0.001
	Colorectal versus other	0.379 (0.214–0.671)	<0.001
	Lung versus other	1.407 (0.767–2.579)	0.270
	Prostate versus other	0.209 (0.073–0.599)	0.004
	Other genitourinary versus other	1.260 (0.652–2.433)	0.492

*BMI* body mass index, *ADL* activities of daily living, *IADL* instrumental activities of daily living, *CIRS* Cumulative Illness Rating Scale, *MMSE* mini-mental state examination, *ECOG* Eastern Cooperative Oncology Group, *Cont. Var.* continuous variable

**Fig. 1:**
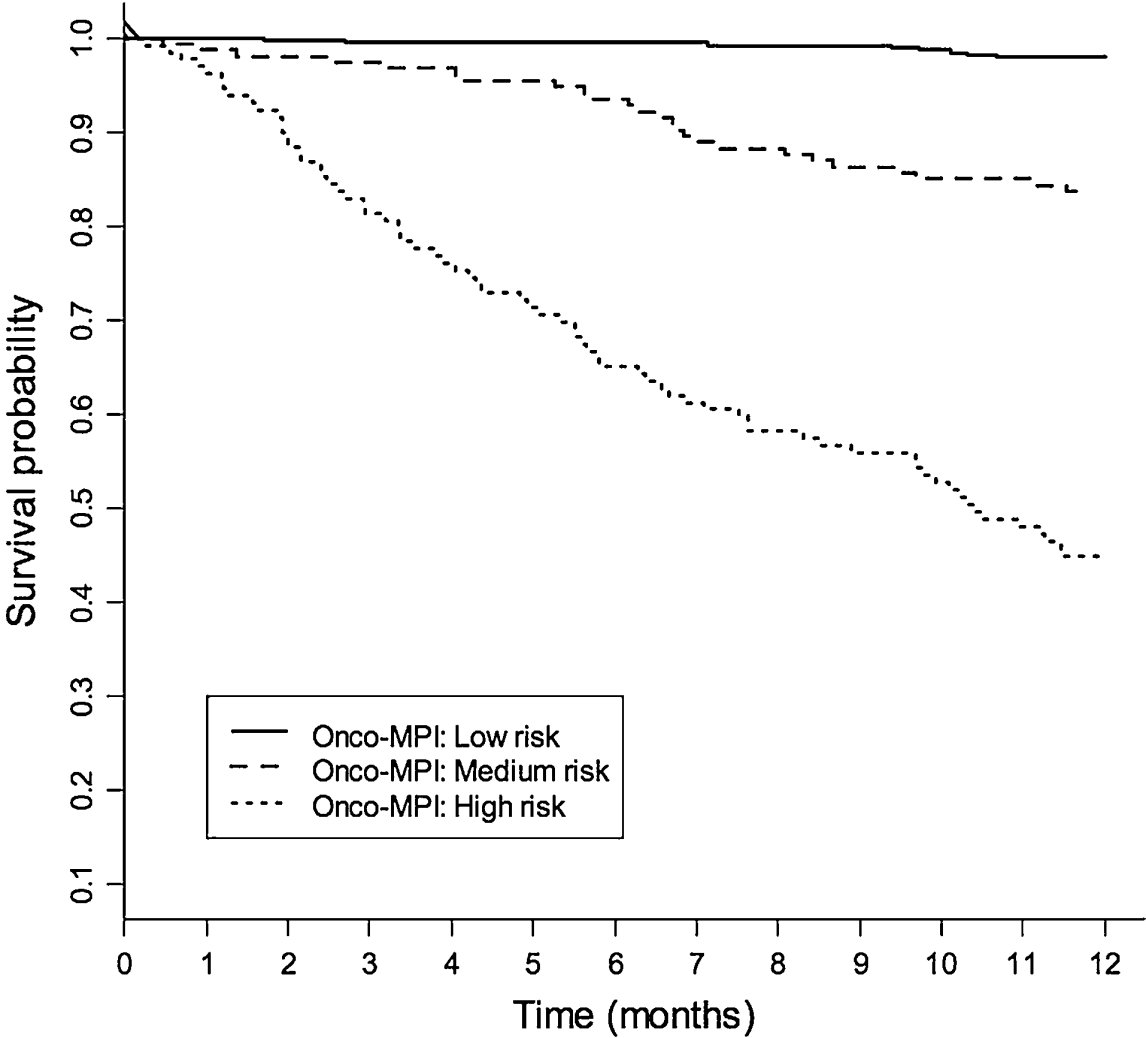
Kaplan-Meier survival curves, within 1 year of follow-up, according to the three Onco-MPI risk score categories (low risk, medium risk and high risk)

The Onco-MPI score had a good discriminatory power, yielding a C-statistic of 0.869 (95 % CI 0.841–0.897) and a good calibration measure (HL *p* value = 0.854). Three risk score categories were estimated for Onco-MPI score using RECPAM method, according to the following cutoffs: 0–0.46 (low risk), 0.47–0.63 (moderate risk) and 0.64–1 (high risk). A significant difference in mortality rates was observed for Onco-MPI low risk compared to medium- and high-risk patients (2.1 vs. 17.7 vs. 80.8 %, respectively, *p* < 0.001). Kaplan–Meier survival curves for one-year mortality risk, according to the three risk score categories (low risk, medium risk and high risk), are shown in Fig. 1.

## Discussion

In the present study, the cancer-specific Onco-MPI appeared to be a highly accurate and well-calibrated prognostic tool for one-year mortality in older cancer patients that can be useful for defining homogeneous prognostic categories and clinical decision making in this age group. Indeed, therapeutic decisions in elderly cancer patients are not fully informed unless heterogeneity of the aging process is taken into account. Actually, some forms of CGA have been successfully used to establish individualized treatment plans of treatment (Caillet et al. 2011) and in defining risk of toxicity from treatments in older cancer patients (Hurria et al. 2011; Extermann et al. 2012). Thus, current clinical guidelines for cancer in older age recommend to implement the CGA methodology (Extermann et al. 2005; Biganzoli et al. 2012; Droz et al. 2010; Pallis et al. 2010) in order to determine the residual biological, psychological and functional capabilities of the older patients, i.e., the grade of frailty (Baijal and Periyakoil 2014; Hamaker et al. 2012), for developing a personalized plan for treatments and interventions. Indeed, whatever the definition and methodology used to evaluate frailty, frail patients have a higher mortality compared to non-frail patients. In a previous study, we showed that frail hospitalized patients, being treated despite poor conditions, had poor outcome (Basso et al. 2008). Furthermore, frail lymphoma patients had same outcome whether they were treated with active modified oncological treatment or palliative care (Tucci et al. 2009). The prognostic evaluation of life expectancy emerges thus as a key factor by which pros and cons of active oncological treatment must be weighted, both in the adjuvant setting and in the metastatic setting. Prognosis could be also fundamental for balancing the harm–benefit and cost–benefit ratios in situations of uncertainty when prescribing high-cost drugs or treatments requiring multiple admissions with potential impact on quality of life. In recent years, some prognostic scores have been proposed, but none of these was based on information collected by a standardized CGA (Yourman et al. 2012; Pilotto et al. 2015; Baijal and Periyakoil 2014). The MPI has been previously validated in older hospitalized patients suffering from major diseases, including several types of cancer (Pilotto et al. 2012a, b, c; Giantin et al. 2013), with a significant higher predictive power than other widely used frailty indexes (Bellera et al. 2012). In this cohort from an outpatient setting, we developed a modified MPI that included, compared to the originally MPI, the ECOG performance status (instead of the Exton-Smith scale), the MMSE (instead of the Short Portable Mental Status Questionnaire, SPMSQ) and the BMI (instead of the Mini Nutritional Assessment, MNA) to evaluate functional, cognitive and nutritional status, respectively.

Notably, we further included in the prognostic model both patients’ cancer site and cancer stage variables, along with the main MPI domains, due to their clinical relevance on the mortality risk prediction. For tumor sites, some biases can have been introduced since breast cancer was the prevalent type (46.5 %) with early stages of disease being more represented for this tumor site, whereas other tumor sites, i.e., lung cancer, were far less prevalent, with higher stages of disease at first access. Globally, 75 % of patients received active oncology treatment whose impact on survival was not significant. Therefore, considering multidimensional aggregate information may go beyond the heterogeneity of the sample related to diverse cancer sites and stages, and the variety of treatments used (endocrine agents, several types of chemotherapy regimens).

While screening tools based on abbreviated CGA showed high sensitivity with low specificity in predicting mortality (Smets et al. 2014; Bellera et al. 2012), CGA-based prognostic scores similar to the Onco-MPI in older cancer patients have been poorly investigated. Recently, a study conducted on 249 older Asian cancer patients by using CGA items to build a prognostic nomogram based on six clinical laboratory items demonstrated a relatively low predictive accuracy for one-, two- and three-year overall survival with a C-index value of 0.71 (Kanesvaran et al. 2011). The Onco-MPI was developed on more than 600 patients using some CGA-based items which can be easily implemented in routine oncological practice to drive treatment decisions. A time horizon of 1 year was chosen because we focused on a short-term mortality risk prediction, which is particularly helpful to give systemic oncological treatment or not in many cancers of the older people.

In this study, ECOG performance status was significantly associated with mortality, thus confirming the well-known prognostic role of this tool in oncology. Moreover, both ADL and IADL in our model were significantly associated with mortality in univariable analysis, and the loss of significance in the multivariable analysis probably was related to interaction with other considered domains (Extermann et al. 1998). The presence and number of severe comorbidities have also been found to be related to poor prognosis in the present study. Indeed, comorbidity has been consistently proven to be associated with worse survival in older cancer patients (Piccirillo et al. 2004). This has been shown to be independent of ECOG performance status (Firat et al. 2002) and functional status (Extermann et al. 1998). Weight loss or a low BMI was associated by reverse epidemiology with an increased risk of mortality in older age (Landi et al. 2000), a finding confirmed also in our cohort of cancer patients Furthermore, cognitive status showed a strong prognostic value in our older cancer patients, confirming earlier findings suggesting dementia as an independent prognostic factor for survival in older subjects (Wolfson et al. 2001). Depression has been also proposed as a predictive factor of mortality in older cancer patients with controversial findings (Satin et al. 2009). In our study, the presence of depression was not associated with survival, and therefore it was not considered among variables for building the Onco-MPI. In both the geriatric and oncology literature, social isolation has been linked to an increased risk of mortality (Kroenke et al. 2006). In our model, the presence of a caregiver was unexpectedly related to worse survival, probably because in an outpatient setting, those requiring a caregiver could have been the most vulnerable ones.

The calculation of Onco-MPI can be easily performed through an excel file in which the value of each domain (age, sex, BMI, ADL, IADL, ECOG PS, MMSE, number of severe comorbidity, cancer stage, tumor site, number of drugs, the presence of caregiver) is multiplied by the coefficient which is the weight for Onco-MPI (reported in Table 3).

From a practical standpoint, we can take the example of an 80-year-old woman with stage III colorectal cancer, PS 1, ADL 6/6, IADL 5/8, BMI 28, good cognitive status (MMSE ≥ 24), two severe comorbidity, five drugs, with caregiver present; this patient has an Onco-MPI score of 0.44, which means that she is in the lower Onco-MPI score, with an estimated risk of mortality at 1 year of 2.1 %. The same woman, but having a bad cognitive status (MMSE < 24) and BMI 19, has an Onco-MPI score of 0.54, which means that she is in the intermediate risk group, with an estimated one-year mortality of 17.7 %. If this patient had ADL 5/6, her Onco-MPI would be 0.72, which corresponds to higher risk of mortality at 1 year, estimated to be more than 80 %. While adjuvant treatment may be discussed in the first case and carefully evaluated in the second case, in the third case geriatric assessment and oncoMPI suggest not to consider adjuvant chemotherapy. It is important to notice that the patient was judged as having a PS 1 in all cases, thus confirming that comprehensive geriatric assessment adds information, and in the case of the Onco-MPI adds prognostic information, to simple PS estimation (Repetto et al. 2002).

The main limitations of the present study were the possible selection of more “fit” older cancer patients and the lack of data of the predictive role of the Onco-MPI for mid- and long-term mortality. The Onco-MPI has been built on outpatients; therefore, there is a greater likelihood of the inclusion of less frail patients which may have skewed the results. However, it holds a very good discriminatory power with a C-statistic of 0.87. In fact, despite the lack of accepted criteria to assess the quality of prognostic indices, generally C-statistics for discrimination can be considered good for ranges 0.70–0.79, very good for ranges 0.80–0.89, and excellent for 0.90 or greater (Yourman et al. 2012). Beyond its prognostic ability, the Onco-MPI could also serve as an useful tool for evaluating effectiveness of an intervention in different settings, when changes in Onco-MPI categories that can be obtained may reflect the outcome of the intervention, as recently reported in older patients with late-life major depressive disorder responders and non-responders to antidepressant treatment (Pilotto et al. 2012a, b, c). Finally, the Onco-MPI in the research setting may help to properly classify patients enrolled in clinical trials, selecting more homogeneous subgroups of patients. However, the Onco-MPI warrants external validation, which is already underway, and proof of ability to predict mortality with longer follow-up.
